# Carapace Morphology Variations in Captive Tortoises: Insights from Three-Dimensional Analysis

**DOI:** 10.3390/ani14182664

**Published:** 2024-09-13

**Authors:** Ebru Eravci Yalin, Ozan Gündemir, Ebuderda Günay, Ezgi Can Vatansever Çelik, Sokol Duro, Tomasz Szara, Milos Blagojevic, Bektaş Sönmez, Mihaela-Claudia Spataru

**Affiliations:** 1 Department of Surgery, Faculty of Veterinary Medicine, Istanbul University-Cerrahpasa, Istanbul 34320, Türkiye; ebrueravci@gmail.com; 2 Department of Anatomy, Faculty of Veterinary Medicine, Istanbul University-Cerrahpasa, Istanbul 34320, Türkiye; 3 Department of Wild Animal Diseases and Ecology, Faculty of Veterinary Medicine, Istanbul University-Cerrahpasa, Istanbul 34320, Türkiye; ebuderda.gunay@iuc.edu.tr; 4 Faruk Yalcin Zoo and Botanical Garden, Kocaeli 41700, Türkiye; ezgi.celik@farukyalcinzoo.com; 5 Department of Morphofunctional Modules, Faculty of Veterinary Medicine, Agricultural University, 1000 Tirana, Albania; durosokol@ubt.edu.al; 6 Department of Morphological Sciences, Institute of Veterinary Medicine, Warsaw University of Life Sciences-SGGW, 02-776 Warsaw, Poland; 7 Department of Anatomy, Faculty of Veterinary Medicine, University of Belgrade, 11000 Belgrade, Serbia; mblagojevic@vet.bg.ac.rs; 8 Suşehri Timur Karabal Vocational School, Sivas Cumhuriyet University, Sivas 58600, Türkiye; bektass@gmail.com; 9 Department of Public Health, Faculty of Veterinary Medicine, Iasi University of Life Sciences, 700489 Iasi, Romania; mspatarufmv@yahoo.com

**Keywords:** geometric morphometrics, tortoises, carapace, anatomy, *Stigmochelys pardalis*, *Sulcata tortoises*, *Testudo graeca*

## Abstract

**Simple Summary:**

This study explored the morphological differences in the carapaces of three tortoise species—Leopard tortoises (*Stigmochelys pardalis*), African spurred tortoises (*Centrochelys sulcata*), and Greek tortoises (*Testudo graeca*)—raised in captivity in Türkiye. Using 3D scanning and geometric morphometric analysis, we identified the vital shape variations and dimensional features across species. Principal component analysis revealed that PC1 and PC3 were significant in distinguishing species, with Leopard tortoises showing higher carapace shapes and African spurred tortoises having shorter carapaces. An allometric effect indicated that smaller tortoises had higher shells. These findings provide valuable insights for taxonomy and clinical practice, emphasizing the need to consider shape variations in tortoise care and species identification.

**Abstract:**

The carapace morphology of tortoises is a crucial characteristic used for species identification, with features such as shell shape, roughness, and color patterns varying among species. Understanding this morphological diversity is valuable not only for taxonomic classification but also for more specialized clinical approaches. This study investigated the morphological differences in the shells of Leopard tortoises (*Stigmochelys pardalis*), African spurred tortoises (*Centrochelys sulcata*), and Greek tortoises (spur-thighed tortoises; *Testudo graeca*) raised in captivity. Using 3D scanners, the carapaces were modeled, and a 3D geometric morphometric method was employed to analyze shape variations and dimensional features, with landmarks applied automatically. Among the species studied, African spurred tortoises had the largest carapace size. Principal component analysis (PCA) identified PC1 and PC3 as critical factors in distinguishing between species based on morphological characteristics. Positive PC1 values, associated with a shorter carapace height, indicated a flatter or more compact shell shape. A higher PC3 value corresponded to a raised shape at the back of the shell, while a lower PC3 value indicated a raised shape at the front. Specifically, Leopard tortoises exhibited a higher carapace shape than the other species, while African spurred tortoises had shorter carapaces. An allometric effect was observed in the carapaces, where smaller specimens tended to be proportionately higher-domed, whereas larger shells displayed a lower height in shape. These findings highlight the significance of shape variations in tortoise shells, which emerge during adaptation and have important implications for taxonomy and clinical practice. Such differences should be carefully considered in veterinary care and species identification.

## 1. Introduction

Tortoises, classified under the family Testudinidae, are a diverse group of reptiles encompassing 60 living species distributed across a wide geographical range. Their habitats span various terrestrial environments, ranging from coastal plains to arid deserts and lush tropical rainforests. Primarily, herbivorous tortoises play a vital role in their ecosystems by influencing plant growth and distribution through feeding habits [1,2,3]. Known for their characteristic slow movement, tortoises rely on their sturdy, clawed feet to traverse their surroundings. This slow pace is compensated for by their remarkable longevity, with some species living well over a century. This longevity and their distinctive shell structure and herbivorous diet make tortoises easily recognizable and iconic representatives of the reptile world [4,5]. Overall, tortoises exemplify the adaptability and longevity of reptiles, serving as essential components of their ecosystems and captivating symbols of nature’s resilience and diversity. 

Among the vertebrates, Testudines possess a distinctive feature: their dorsal vertebrae fuse to create a bony shell, encasing vital organs, shoulders, and pelvic girdles while constraining limb movements, except for the neck and tail regions [6,7]. Therefore, the tortoise’s shell is a unique feature among modern vertebrates. It plays a crucial role in shielding the tortoise from predators and environmental hazards [8,9,10] while providing structural support for its body, particularly for the muscles that enable it to retract its head and limbs into the shell for protection. The shell consists of two parts: the carapace and the plastron. The carapace, the dorsal part of a tortoise’s shell, is a protective covering composed of fused bones covered by a layer of keratinized scales known as scutes [11]. Along the carapace’s midline is a nuchal (cervical) scute, followed by five vertebral scutes, four pairs of pleural scutes located more laterally on each side, and twelve pairs of marginal scutes surrounding the first [12,13].

The shape and structure of the carapace can vary significantly among species of tortoises, reflecting adaptations to their specific environments and lifestyles. Conspecific tortoises may exhibit different growth patterns and corresponding variations in shell development when raised in captivity instead of in the wild [14]. In recent years, research emphasis on turtle ecomorphology has increasingly turned toward investigating the relationship between shell morphology and habitat preferences at broader taxonomic levels. For example, *Terrapene* and *Cuora* exhibit similar shell shapes, indicating convergent evolution in response to shared ecological pressures [15]. *Malacochersus tornieri*, a distinct species, has evolved an extraordinarily flat and flexible shell to adeptly navigate and conceal itself among rock formations in its East African habitat [16]. Accordingly, for several reasons, the turtle shell remains a robust structure for studying the morphological evolution of various traits. Homology among shell elements is readily identifiable within a specific taxonomic group. Additionally, the number of epidermal scutes and bony plates has remained consistent throughout the evolution of the Testudines, offering numerous reference landmarks for accurately depicting morphological variations [17].

Various biological processes contribute to the morphological diversity among individuals or their parts, stemming from disease, injury, mutation, and genetic development [18,19]. Shape analysis is an approach to understanding this morphological diversity [20]. Geometric morphometric methods are commonly employed for shape analysis, utilizing statistical tools to elucidate differences in shape. In this method, anatomical landmark positions on the biological shape are collected, and these anatomical points are then evaluated using principal component analysis to identify the vectorial changes that have the most significant impact on shape analysis [21,22]. Apart from size, location, and orientation, landmarks can be analyzed using various statistical techniques, with variables primarily based on morphology. Geometric morphometry involves the application of landmarks on samples in either two or three dimensions. It is considered one of the most current and effective methods for examining the morphology of structures, providing insights into both the visual and quantitative aspects of structural shape.

In recent years, the application of advanced 3D technology in veterinary medicine, particularly in exotic animal care, has revolutionized external biological structure restoration treatments, such as prosthetics. An illustrative example is the prosthetic reconstruction of a damaged turtle shell using 3D modeling techniques at the Istanbul University-Cerrahpaşa Faculty of Veterinary Medicine Animal Hospital. These innovative approaches enhance animal welfare and underscore the importance of understanding species-specific morphological variations to improve clinical outcomes. This study aimed to contribute to veterinary taxonomy by elucidating the shell shape variations among turtle species commonly kept as exotic animals in Türkiye. For this purpose, we investigated the morphological differences in the shells of Leopard tortoises (*Stigmochelys pardalis*), African spurred tortoises (*Centrochelys sulcata*), and Greek tortoises (spur-thighed tortoises; *Testudo graeca*) raised in captivity. The findings of this research are expected to inform future clinical practices, ensuring species-specific considerations in surgical and prosthetic interventions.

## 2. Materials and Methods

### 2.1. Samples

In this study, 24 tortoises were utilized, comprising 5 Leopard tortoises, 4 African spurred tortoises, and 15 Greek tortoises. All specimens used in this study were adults, and their sexes were known (the curvature of the carapace differs between males and females: females typically have a more steeply curved carapace with a greater absolute shell height, while males have a flatter carapace with a smaller absolute height) [23]. The samples were sourced from both Bursa Zoo and Faruk Yalcın Zoo. An essential prerequisite for this study was that all turtles used were healthy and did not exhibit any pathological conditions in their carapaces.

### 2.2. Modeling

Tortoise carapaces were examined in this study, and a Shining 3D EinScan Pro 2X 3D scanner (Shining 3D, Hangzhou, China) was employed for 3D data collection. Specifically, HD scanning mode was utilized to capture detailed images of the carapaces using the device’s manual scanning mode. The scanning speed was set at 20 frames/s, and the dot distance was configured to be up to 0.2 mm, ensuring high-resolution scans. Tortoises were scanned indoors to mitigate the glare caused by sunlight during the scanning processes. Upon completion of the scanning processes, the obtained data were processed using EXScan Pro (v4.0.0.4) software for mesh operations, and the resulting models were saved in PLY format for further analysis.

The Shining 3D EinScan Pro 2X 3D scanner, previously used to scan complex and delicate biological structures such as the human face, provided fast and reliable results in this study [24,25]. The same person operated the manual scanning mode from a distance of 20 cm. When the distance was either greater or smaller than the optimal range, the warnings given by the software were taken into account, and each scan took an average of 3 min. During the scan, obstacles were placed in front of the tortoises to restrict their movement. Shots that showed movement during the scan were repeated. A study protocol was signed with the zoos for using the animals in this study.

Three-dimensional scanners capture (Shining 3D EinScan Pro 2X 3D scanner, Shining 3D, Hangzhou, China) detailed surface information of objects using a range of sensors and technologies. These devices employ lasers or lights to scan the object from multiple angles, creating a comprehensive digital representation. (The light source of the device used in this study was an LED.) In essence, 3D scanners generate a ‘photo’ of the object in three dimensions. The collected data are then processed by computer technology to create a virtual 3D model of the object, which can be used for various applications such as modeling, analysis, and visualization.

### 2.3. Dataset

**Figure 1:**
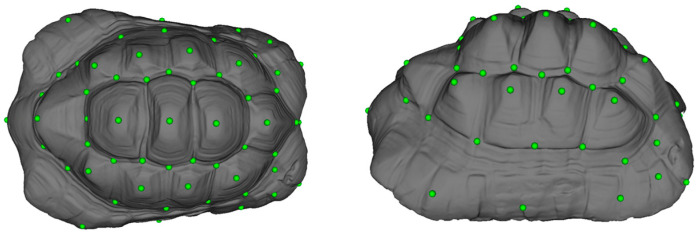
Landmarks.

In this study, 3D geometric morphometric analysis was conducted, starting with creating a draft landmark set. The PseudoLMGenerator module within the Slicer program (version 5.2.2) was utilized for this purpose. A female Leopard tortoise of average size was selected as the reference specimen to create the landmark set. Points were evenly distributed on both sides of the carapace using a mid-sagittal plane. The spacing tolerance was set at 8%, and a template was generated based on this configuration. The template geometry dictated the shape of the estimated sampling template. Landmarks were then marked on the surface of the carapace. Subsequently, the regularity of the sampling was enhanced, resulting in the final landmark set (as shown in Figure 1 with green points). As a result of all operations, a total set of 59 landmarks was obtained.

This study employed automated landmarking through point cloud alignment and correspondence analysis (ALPACA) to process the initial draft landmark set across all other 3D models. This was achieved using the ALPACA module within the Slicer program (version 5.2.2) [26]. The draft landmark set was automatically applied to all samples, streamlining the landmarking process and ensuring consistency across the dataset.

### 2.4. Statical Analysis

The morphological variation in tortoise carapaces was studied in terms of size and shape. Size was quantified using centroid size, calculated as the square root of the sum of the squared distances between all landmarks and the object’s centroid. Shape analysis was conducted using Procrustes distance values. Both centroid size and Procrustes distance values were obtained using the SlicerMorph module in the 3D Slicer program (version 5.2.2) [27].

SlicerMorph was employed to perform a Procrustes fit, which removes variations in scale, position, and orientation, and to identify potential outliers that may have resulted from digitizing errors [27]. Principal component analysis (PCA) based on Procrustes variables was then used to explore the morphological affinities among individuals. Initially, the effect of sex on overall shape was examined [27]. MANOVA results indicated that sex had no significant impact, so all samples were analyzed collectively using the PAST statistics program [28].

The analysis identified the main axes (principal components) along which carapace shape variation occurred. These components were plotted to visualize the variation in carapace shapes among the different species. The principal components’ extreme positive and negative values were visualized using 3D models of the carapaces.

The influence of size on PC1 and PC2, which explained the most variation in shape, was examined to assess the allometric effect. Multivariate regression analysis was conducted using the PAST statistics program to determine the relationship between size and shape variation [28].

## 3. Results

**Figure 2:**
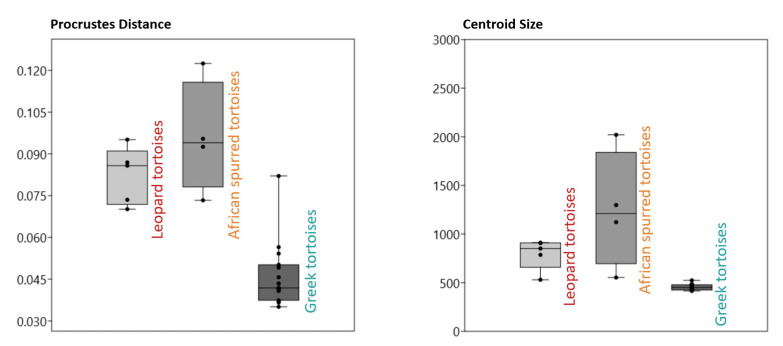
Procrustes distance and centroid size values of tortoise species.

The Procrustes distance and centroid size distributions of the species are depicted in Figure 2. The Procrustes distance illustrates the variation in individuals in terms of the average shape. Considering the large number of specimens, the effect of the Greek tortoise sample on the average shape was expected to be greater than that of the other species. Consequently, the Procrustes distance values for Greek tortoises were lower than those for the other species. However, one specimen of Greek tortoise exhibited a different variation than the other Greek tortoise specimens.

According to the centroid size results, the largest specimen was an African spurred tortoise, while the Greek tortoises were the smallest. Size variation was more significant among African spurred tortoise specimens than among other species.

**Figure 3:**
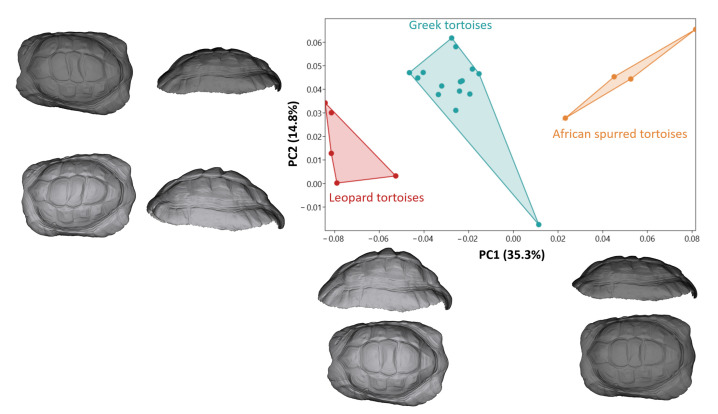
Principal component analysis scatter plot comparing carapace morphology for tortoise species. Models describing carapace shape between the negative and positive (dark color) values of PC1 and PC2 from lateral and dorsal views.

Principal component 1 (PC1) explained 35.3% of the total variation, indicating its significant role in capturing the shape differences among species (Figure 3). PC2, on the other hand, explained 14.8% of the total variation, further contributing to the characterization of species-specific shape variations. The distinct PC1 values observed among species suggest that it may be a critical factor in discriminating between them based on their morphological features.

Leopard tortoises stood out with their low PC1 values, suggesting a unique shape profile. In contrast, African spurred tortoises exhibited the highest PC1 value, indicating a distinct shape pattern within the dataset. 

The observed differences in carapace morphology along the PC1 and PC2 values reflect the intricate variations in shape that the tortoises exhibit. At low PC1 values, tortoises exhibit a more elevated shell profile. Conversely, positive PC1 values, associated with a shorter carapace height, might indicate a flatter or more compact shell shape. The contrasting carapace widths at negative and positive PC1 values suggest differences in the overall width and curvature of the front edge of the shell. A narrower front edge at negative PC1 values might indicate a more pointed shape, while a wider front edge at positive PC1 values could suggest a broader and more rounded frontal profile. Moving to PC2, the downward curvature of the back of the carapace at negative values indicates a more pronounced arch or slope toward the rear of the shell. With these results, Leopard tortoises exhibited a higher carapace shape than the other species. African spurred tortoises exhibited a more flattened carapace shape than the other tortoise species.

**Figure 4:**
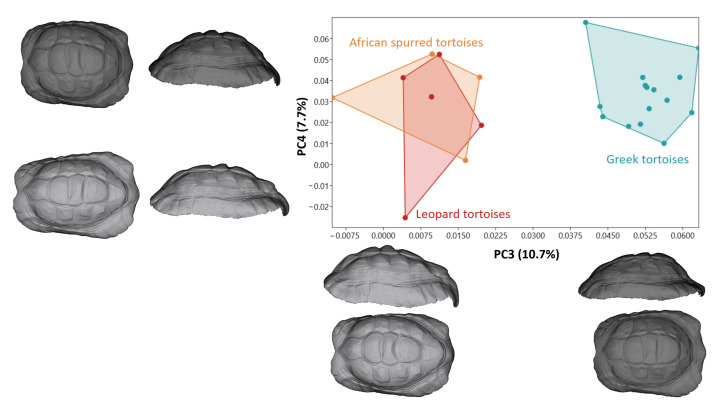
Principal component analysis scatter plot comparing carapace morphology for tortoise species. Models describing carapace shape between the negative and positive (dark color) values of PC3 and PC4 from lateral and dorsal views.

Although PC3 and PC4 together explained 18.4% of the total variation, in the PC3 values, Greek tortoises were distinctly separated from the other species (Figure 4). A higher PC3 value corresponded to a raised shape at the back of the shell, while a lower PC3 value indicated a raised shape at the front. In Greek tortoises, the front of the shell was higher, whereas in Leopard tortoises and African spurred tortoises, the back of the shell was higher than the front.

**Figure 5:**
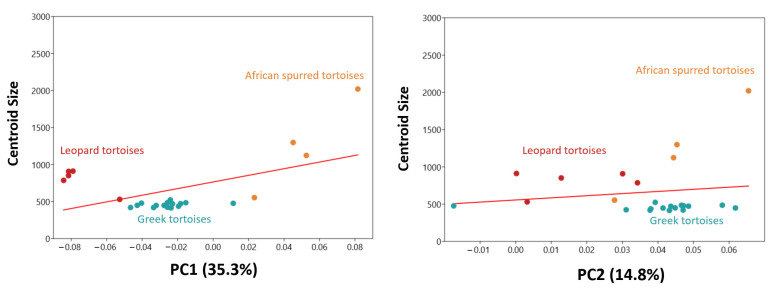
Effect of PC1 and PC2 values on centroid size.

The analysis of the interaction among PC1, PC2, and centroid size provided further insights into the relationship between tortoise carapace shape and size (Figure 5). While PC2 (F: 0.48856; P:0.492) did not significantly affect shape, PC1 (F: 7.2292; P: 0.013) demonstrated a clear influence. According to these results, smaller specimens exhibited shells with greater height, while larger shells tended to have more depressed carapaces.

## 4. Discussion

This study examined the shape variation of exotic land tortoises in Türkiye, focusing on clinical and taxonomic differences between species. Principal component analysis (PCA) revealed that PC1 and PC3 were crucial for distinguishing species based on morphological features. PC1 effectively separated all species from each other, while PC3 specifically distinguished Greek tortoises from the others. A significant allometric effect was observed in PC1, indicating that size-related shape changes contribute to the morphological differences among species. These findings suggest that the shape variations in tortoise shells, which arise during adaptation, have essential implications for taxonomy and clinical practice, where such differences should be carefully considered.

This observation aligns with Dosik and Stayton’s study, where larger tortoise species (e.g., African spurred tortoises) displayed a distinct distribution pattern from that of other species [29]. In the survey by Dosik and Stayton, the size and shape in tortoise shell evolution were examined, confirming shape differences between these species and the hypothesis that various factors govern shell shape in tortoises. They also stated that shell shapes associated with low scores on PC3 might be particularly typical of older individuals of giant island turtles, which lack natural predators. This suggests an evolutionary advantage for specific shell shapes in populations with reduced predation pressure. The results of this study are similar to those of Dosik and Stayton [25]. The tortoise species exhibiting larger body sizes (African spurred tortoises) in this study exhibited differences from other species in terms of shape variation, particularly in the PC1 results, which explained the most variation. African spurred tortoises showed a shape distribution that differed from that of other species. 

Studies have suggested that turtle shell shapes undergo morphological changes during adaptation [29,30]. Additionally, research by Semaha [31] expanded the understanding of shell size in Greek tortoises, revealing differences in size patterns across geographical ranges. These findings indicate that various factors, including environmental conditions, can influence turtle shells. Understanding the interaction between these factors and shell morphology can provide insights into the morphological differences observed in turtle shells. Although the sample size in this study was limited, it focused on examining the shape variations in the shells of turtles living under conditions different from those to which they are accustomed. While we cannot definitively determine which stage of the adaptation process these samples represent or whether they have completed it, the 3D images obtained in this study can be electronically stored. These reference data can be used in future research for the same geographic region or can serve as an essential resource for examining shell variations in the same species across different geographical areas.

In recent years, 3D image reconstruction and 3D printing technology have become increasingly prominent in veterinary medicine, serving various purposes such as for producing custom implants, prosthetics, and orthotics [32,33,34]. These technologies offer several advantages, including creating patient-specific models that are highly realistic and cost-effective and present minimal microbial risk, making them particularly attractive in veterinary clinical practice and education [32,35]. The use of 3D printing in veterinary medicine is beneficial for its economic advantages and ability to facilitate customized treatments. These models are typically generated using computed tomography (CT) and magnetic resonance imaging (MRI) data. However, 3D scanners offer a more practical alternative for structures like turtle shells. Unlike CT and MRI, which involve X-rays and may raise ethical concerns, 3D scanning is quicker and non-invasive [36]. In this study, the shells of various exotic turtles in Türkiye were scanned in 3D and digitally modeled. Advanced 3D scanning technology made the fieldwork highly efficient, with each sample taking approximately three minutes to scan. This method, which completes imaging quickly and avoids ethical issues, can be considered the most suitable technique for modeling turtle shells with current technology.

In the context of potential turtle shell surgeries, 3D scanning can capture the shell’s undamaged side. A prosthesis can be created by reverse engineering the missing part in a computer environment. Like those obtained in this study, reference data can be clinically valuable for more considerable tissue losses.

One limitation of this study is the relatively small sample size for each group of tortoises. Specifically, we examined 24 tortoises, namely, 5 Leopard tortoises, 4 African spurred tortoises, and 15 Greek tortoises. This limited number of specimens made it challenging to determine if the observed differences in shell morphology were statistically significant. Since there were no more examples of these species in zoos in Türkiye, the scope of this study was limited by the number of individuals we could access. However, the findings still provide valuable reference data and contribute to understanding shell shape variations among these tortoise species. Future research with larger sample sizes would help confirm and expand upon these initial observations.

## 5. Conclusions

In conclusion, our study successfully examined the shape variations in the carapaces of different tortoise species in captivity, including Leopard tortoises, African spurred tortoises, and Greek tortoises. Through our analysis, we found significant differences in carapace morphology among these species, highlighting distinctive shape patterns that distinguish them. Our findings contribute to a better understanding of the morphological diversity within tortoise populations and emphasize the unique characteristics of each species’ carapace shape. These insights can inform future research on tortoise morphology and aid conservation efforts to preserve these iconic species. 

## Data Availability

The data presented in this study are available upon request from the corresponding author.
